# Outcomes of root canal treatments with three different sealers for 120 fractured maxillary fourth premolar teeth in small-to medium-sized dogs

**DOI:** 10.3389/fvets.2024.1382645

**Published:** 2024-05-09

**Authors:** Daehyun Kwon, Dae Sung Yoo, Seong Soo Kang, Kwangsik Jang, Se Eun Kim

**Affiliations:** ^1^MAY Veterinary Dental Hospital, Seoul, Republic of Korea; ^2^Department of Veterinary Surgery, College of Veterinary Medicine and BK21 Plus Project Team, Chonnam National University, Gwangju, Republic of Korea; ^3^Department of Veterinary Public Health, College of Veterinary Medicine, Chonnam National University, Gwangju, Republic of Korea; ^4^Biomaterial R&BD Center, Chonnam National University, Gwangju, Republic of Korea

**Keywords:** tooth fracture, root canal treatment, silicone-based sealer, bioceramic sealer, calcium hydroxide-based sealer, working length, master apical file, dog

## Abstract

**Introduction:**

Tooth fracture is one of the most common traumatic maxillofacial injuries in dogs and cats. For fractures with pulp exposure occurring in functionally important teeth, the literature indicates that root canal treatment (RCT) is an effective therapy option that may be the remedy of choice before extraction. The most commonly reported fractures in the United States involve canine teeth; however, fractures of the maxillary fourth premolars are more common in Korea, where there are many small-and medium-sized dogs. RCT mechanically and chemically removes pulp tissue and bacteria (cleaning and shaping) from the infected root canal, and obturates the root canal with filling material to restore tooth functionality without inflammation. Various techniques, instruments, and materials used in humans have been modified for application in veterinary dentistry.

**Methods:**

This study analyzed the results of RCT of the maxillary fourth premolar in 120 small-and medium-sized dogs (weighing less than 25 kg) using three different sealers (silicone-based sealer, bioceramic sealer, and calcium hydroxide-based sealer) through a simple application of the single-cone technique.

**Results:**

The overall success rate of RCT in maxillary fourth premolars was 90.83%, with 8.33% no evidence of failure (NEF) and 0.83% failure.

**Discussion:**

There were no significant differences between the three different sealers. Furthermore, preexisting periapical lesion (PAL) was reconfirmed as a factor in reducing the success rate of RCT. In addition, the working length and master apical file of each root were analyzed in our study as a novel reference for endodontic veterinarians.

## 1 Introduction

Traumatic tooth injury is a common problem in dogs. Twenty-seven percent of the general population and 72.1% of maxillofacial injury cases are reported to have dentoalveolar problems (1, 2). Complicated crown fractures (CCF) are the most common traumatic dentoalveolar injuries in dogs and cats (3). Considering the functional importance of the various teeth in dogs, the canines, maxillary fourth premolars, and mandibular first molar teeth are classified as “strategic teeth” (4). Therefore, CCF of these teeth can have serious functional consequences. Epidemiologic studies suggest that the canine is the most commonly fractured tooth with pulp involvement (3, 5). Previous studies suggest that the fracture rate of maxillary fourth premolars is significantly higher than that of other major teeth (6, 7). Soukup et al. determined that the majority of traumatic dental injuries occurred in strategic teeth; of the carnassial teeth injured, 92.2% were the maxillary fourth premolars (3).

Root canal treatment (RCT) is an effective treatment option for recovering normal function by avoiding tooth extraction and eliminating inflammation and pain in cases of CCF of strategic teeth with pulpitis and periapical lesions (PAL) (4, 8). In humans, the success rate of RCT is reported to be as high as 95% (8), however, the overall success rate of RCT of multiple teeth in dogs is reported to be 69 to 71% (95 to 96%, including no evidence of failure [NEF]) (8, 9). A recent study indicated that the RCT success rate for canine teeth in dogs was 92.73% (98%, including NEF) (4).

Various cleaning, shaping, and obturation methods that originated from human dentistry have been modified to suit the anatomical tooth structure of dogs in veterinary dentistry (5, 8, 10–14). Theoretically, single-cone techniques in human studies have a slightly lower rate of solid filling of the root canal than thermoplasticized gutta-percha (GP) systems or cold lateral compaction, resulting in a 4–5% increase in failure rate compared to other methods. However, the single-cone technique is the fastest and easiest to learn (15). If the GP closest to the shape of the root canal is chosen, most of the apical third, can be filled with GP and minimal sealer.

Several *in vitro* studies that compared the apical sealing abilities of different sealers (AH Plus, GuttaFlow 2 or Resilon [a thermoplastic synthetic polymer-based endodontic material]) in canine teeth of dogs reported that none of the sealers created a perfect fluid-tight apical seal, and there was no statistically significant difference in microleakage (16, 17). There has been a comparative *in vivo* analysis of the sealing ability of three endodontic sealers in laboratory dogs (18). However, to the best of our knowledge, no studies have evaluated the effects of different sealers on RCT success in dogs. Furthermore, most studies have focused more on canine teeth, leaving a critical gap in the literature on the RCT success of maxillary fourth premolar teeth.

This retrospective study evaluated the outcomes of root canal treatment using an engine-driven rotary nickel-titanium (Ni-Ti) file system and single-cone obturation techniques for CCF and complicated crown root fractures (CCRF) of the maxillary fourth premolars of small-to medium-sized dogs. Three different types of sealers were used, including a silicone-based sealer (GuttaFlow 1^®^ or GuttaFlow 2^®^, COLTENE, Altstätten, Switzerland), a bioceramic sealer (One-Fil^®^, Mediculus, Cheongju-si, Republic of Korea), and a calcium hydroxide-based sealer (Sealapex™, Brea, CA, United States), and their outcomes were analyzed. The working length (WL) and master apical file (MAF) size of each root determined in the RCT of the maxillary fourth premolars of 120 small-and medium-sized dogs were evaluated.

## 2 Materials and methods

### 2.1 Medical records and case selection criteria

The medical and dental records of the MAY Veterinary Dental Hospital were searched to identify cases of fractured maxillary fourth premolars in dogs with RCT over the last 10 years (2013–2022). Only dogs that underwent at least one follow-up with intraoral radiography under general anesthesia at a minimum of 3 months after the initial RCT were included in this study. The most recent image was evaluated in dogs that returned for more than one visit during the study period. Teeth with severe periodontal disease such as stages 3–4 periodontal disease and/or furcation stages 2–3, were excluded from this study. Sex, spay/neuter status, age at treatment (months), breed, and body weight were recorded. Medical and dental records were reviewed to determine the condition of the affected teeth, type of sealer used, and the time of follow-up examinations. Intraoral radiographs acquired before and after RCT and during all follow-up examinations were reviewed.

### 2.2 Radiographic evaluation and diagnostic criteria

Two veterinarians with practices limited to veterinary dentistry (DVM. Kwon and Prof. Kim) evaluated all the digital radiographs on a medical-grade computer screen (Kodak Carestream RVG 6200, Carestream Dental LLC, Atlanta, GA, United States and CR7 Vet Digital X-ray, iM3 Inc., Vancouver, WA, United States). PAL was defined as the widening of the periodontal ligament space, and the widest point of the periodontal ligament space was measured. If the periodontal ligament space was wider than twice the width of the periodontal ligament space at other sites on the radiographic images obtained before and after RCT and at each follow-up, it was recorded as “widening of the periodontal ligament space” (4, 8, 9). The presence or absence of all PAL was also recorded.

Outcomes were categorized as success, NEF, or failure according to the endodontic outcome guidelines established by the European Society of Endodontology (19). Treatment was considered successful if the periapical periodontal ligament space was normal, or if the PAL disappeared and preoperative external inflammatory root resorption (EIRR), if present, had stabilized. If the preoperative EIRR had stabilized and the existing PAL remained the same or decreased in size but had not completely disappeared, the treatment was judged as NEF. If the PAL developed after RCT or the EIRR developed and the PAL became larger than before treatment, it was judged as a failure.

### 2.3 Measurements during the root canal shaping process

The WL of each of the three root canals (mesiobuccal, mesiopalatal, and distal roots) and the MAF size were recorded during the RCT using a 0.04 taper engine-driven rotary Ni-Ti file system (Dentsply Sirona, Charlotte, NC, United States).

### 2.4 Root canal treatment procedure

**Figure 1:**
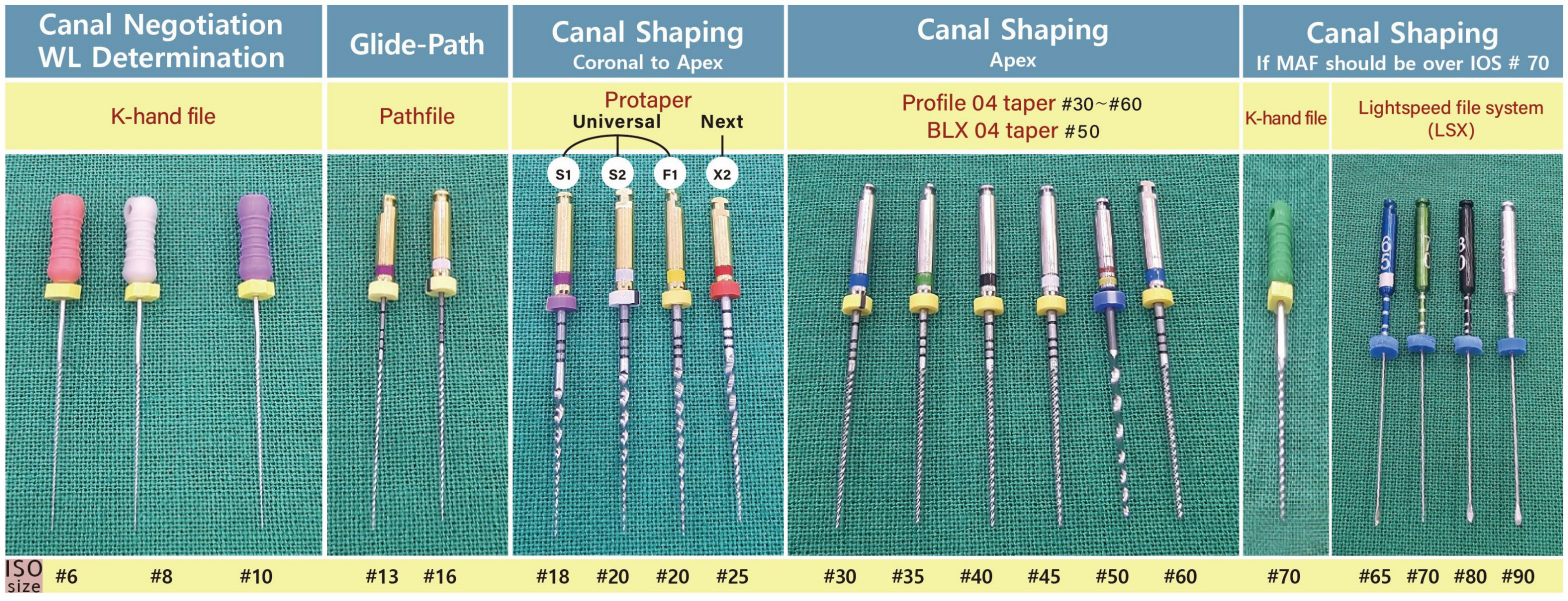
Files in order of use from canal negotiation to determination of MAF.

Direct access to the distal root canals and transcoronal access to the mesial root canals was created, and the orifice was secured in a manner widely accepted in veterinary dentistry. A #10 K-hand file was used to negotiate the canal and determine the WL. If the canal was too narrow for the #10 K-file to enter, an #8 or #6 K-file was utilized to traverse the WL and extend the canal to the width of the #10 K-file. Subsequently, the root canal was shaped using an engine-driven rotary Ni-Ti file system until the MAF was determined. After operating the #10 K-hand file, the Pathfile was extended to the root end in the order of #13 and #16 to secure the glide-path. Next, Protaper Universal S1 and S2 files were applied to shape the coronal and middle parts of the root canal so that subsequent files could be easily used to access the apex. Protaper Universal F1 and Protaper Next X2 were used to shape the apical one-third of the root canal. We used 0.04 taper Profiles to shape the root apex from #30 until the MAF was determined. Because the Profile does not provide #50 and #55 files, we used the BLX 0.04 taper #50 file of FKG (Cret-du-locle, Switzerland) before introducing the #60 Profile. If the MAF could not be established at #60, a K-hand file or a Lightspeed file system of Sybronendo (Glemdora, CA, United States) was used for a larger MAF because the Profile was unavailable over #70 in Korea. The number of root canals with a MAF > #70 was limited. The file used in root canal negotiation to determine MAF are shown in Figure 1.

Sodium hypochlorite (5.25% NaOCl) was applied to the prepared canal to irrigate the cavity between each filing step and the canal was flushed with sterile saline. In addition, 17% ethylenediaminetetraacetic acid (EDTA) was placed in the canal for 1 min and rinsed with sterile saline, and the canals were dried with absorbent paper points.

The single-cone obturation technique was used in this study. The GP point corresponding to the determined MAF was applied to the canal using a sealer, cut at the orifice, and vertically compressed. Non-heated silicone-based sealers, bioceramic sealers, or calcium hydroxide-based sealers were used.

Silicone-based sealers have been widely used in veterinary dentistry for a long time, whereas bioceramic sealers have been introduced relatively recently. Calcium hydroxide-based sealers have been widely used in human dentistry in Korea. As this was a retrospective study, it was not possible to compare the effects of a wide variety of sealers.

The intermediate layer was restored with a glass ionomer, and the access site was sealed with a composite resin.

### 2.5 Follow-up evaluations

For follow-up examinations after the initial RCT, the dogs were anesthetized and intraoral radiographs were obtained. The first follow-up was performed at least 3 months after treatment, and multiple examinations were recommended. After the standard 3 months, 99 teeth were followed up at least once, 18 teeth were followed up twice, and 6 teeth were followed up three times. However, only 120 out of 123 teeth were analyzed in this study because the evaluation was restricted to groups of small-to medium-sized dogs weighing less than 25 kg. For the statistical analysis, the time of the last follow-up was categorized as follows: 3–6 months, 7–12 months, 13–24 months, 25–36 months, 37–48 months, 49–60 months, and > 61 months.

### 2.6 Statistical analysis

Utilizing an ordinal scale to categorize the outcomes of RCT into failure, NEF, and success, a Bayesian multivariate ordinal regression analysis was conducted to examine the relationship among various independent variables, namely, age, weight, number of preexisting PAL, and sex across a sample of 120 patients.


$${RCT}_{i}∼\mathrm{Ordered}-\mathrm{logit}\;\left(\phi_{i},\kappa \right)$$

$$\log\frac{\mathrm{Pr}\left(Rct_{i}\leq k\right)}{1-\mathrm{Pr}\left(Rct_{i}\leq k\right)}=\alpha_{k}-\phi_{i}$$

$$\phi_{i}=\beta_{1}\mathrm{Age}+\beta_{2}\mathrm{weight}+\beta_{3}\mathrm{PAL}+\beta_{4}\mathrm{Sex}$$

In the Bayesian regression framework, the posterior marginal distribution of each regression coefficient was estimated to elucidate the directional association of individual variables with RCT outcomes, while accounting for the influence of other covariates. Specifically, if the mode of the posterior marginal distribution for a given regression coefficient was positive, it indicated that higher values of the associated variable contributed to increased odds of falling into a more favorable outcome category, such as “success,” as opposed to a less favorable outcome category.

Furthermore, a multivariate linear regression model was employed to identify the relationships among several independent variables, namely, age, sex, weight, number of roots with preexisting PAL, type of sealer, and the outcome variables under study. This analytical approach quantified the influence of each variable while adjusting for effect confounders.

## 3 Results

One hundred eighty-two dogs were subjected to RCT for fractured maxillary fourth premolars at the MAY Veterinary Dental Hospital over 10 years. However, only 122 dogs, corresponding to 123 treated teeth, returned for at least one follow-up examination, conducted at least 3 months post-RCT. Our investigation was exclusively confined to the analysis of 120 teeth of small-to medium-sized dogs weighing less than 25 kg. The characteristics of the study population are presented in Table 1. The mean age and weight were 5.45 years and 9.53 kg, respectively. Males and females were almost equally represented (males, 46.6% and females, 53.3%). Twenty-four out of 120 teeth had preexisting PAL in one to three roots.

**Table 1:** Summary of patients (*N* = 120) that received root canal treatment.

Variable		Value	Count
Outcomes of RCT	Success	109 (90.83%)	*N* (rate)
	No evidence of failure	10 (8.33%)	
	Failure	1 (0.83%)	
Sex	Male castrated	56 (46.7%)	
	Female	12 (10%)	
	Female spayed	52 (43.3%)	
Type of sealer	Silicone-based sealer	67 (55.8%)	
	Bioceramic sealer	35 (29.2%)	
	Calcium hydroxide-based sealer	18 (15.0%)	
Fractured tooth	Right maxillary fourth premolar	59 (49.2%)	
	Left maxillary fourth premolar	61 (50.8%)	
Type of fracture	CCF	74 (61.7%)	
	CCRF	46 (38.3%)	
Independent variable	Follow-up duration (months)	11.65 ± 4.1	Mean ± SD
	Age (years)	5.45 ± 2.24	
	Weight (kg)	9.53 ± 4.59	

RCT, root canal treatment; CCF, complicated crown fracture; CCRF, complicated crown-root fracture; SD, standard deviation.

**Figure 2:**
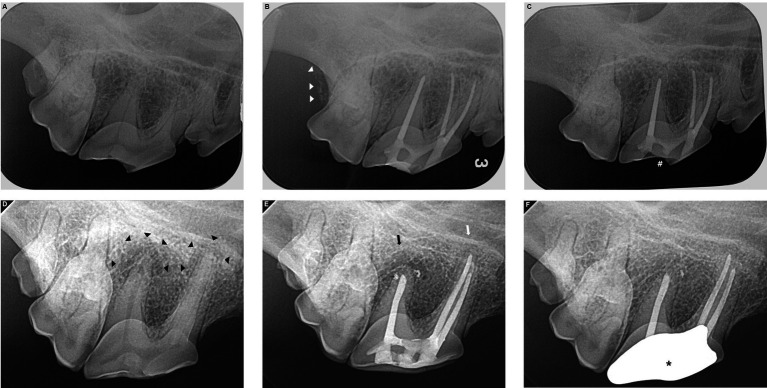
Root canal treatment outcomes with silicone-based sealer **(A–C)** or bioceramic sealer **(D–F)**. Complicated crown root fracture (CCRF) of the right maxillary fourth premolar in a 14.7 kg neutered male Cardigan Welsh Corgi **(A–C)**. Radiography performed before **(A)** and immediately after root canal treatment (RCT) with a silicone-based sealer indicated good shaping and obturation with no voids in the root canals **(B)**. Right maxillary second molar tooth was extracted after RCT because of its periodontitis (white arrow heads). Follow-up radiographs at 22 months postoperatively demonstrate that the apical areas of all three roots are well maintained with no identified lesions **(C)**. Resin composite restored on the fractured surface has been fallen out (#). Complicated crown fracture (CCF) of the right maxillary fourth premolar in a 19.4 kg neutered male Shiba Inu **(D–F)**. A periapical lesion (PAL) is identified around the apex of the distal and mesiopalatal roots (black arrow heads). RCT was performed using a bioceramic sealer **(D)**. The first follow-up was performed 5 months after surgery **(E)**, and the PAL in the mesiopalatal root has disappeared (white arrow). The PAL in the distal root is somewhat smaller (black arrow). An overfilled bioceramic sealer is observed (small strikes). At the 11-month postoperative follow-up **(F)**, the PAL around the distal root has disappeared, and the overfilled bioceramic sealer has absorbed. The full metal crown installed after confirming no evidence of failure at the first follow-up is the most radiopaque (large strike).

Of the 120 examined teeth, 27 were assessed within the 3–6 months, and 50 were evaluated between 7 and 12 months post-treatment. Teeth that were followed up within 3–12 months after treatments accounted for 64.16% of all teeth. Notably, 96 teeth underwent a single follow-up, 18 were evaluated twice, six were subjected to three follow-up examinations, and three were finally assessed after 61 months. The values according to sex, fractured tooth site, type of fracture, sealers, and overall outcomes of the RCT were summarized, and follow-up duration, age, and weight were calculated as means (Table 1). The two cases show outcomes associated with RCT using silicone-based and bioceramic sealers (Figure 2).

When we analyzed the weight of the dogs in the RCT in 5 kg increments, the 5.1–10 kg weight group was the largest, followed by the 10.1–15 kg group (Table 2). These two groups collectively accounted for 90 out of the 120 teeth (75%). Notably, only one tooth was categorized as failure, in a dog in the 2.5–5 kg weight group. Comparing the outcomes of overall RCT and RCT of teeth with preexisting PAL, teeth with preexisting PAL had significantly lower RCT success rates (Table 3). For teeth exhibiting preexisting PAL, the probability of a successful RCT outcome, as opposed to combined NEF and failure, was 0.43 times higher, translating to a 56% decrease in the likelihood of success (Table 4). Specifically, teeth with an increased number of roots affected by preexisting PAL exhibited a 56% reduction in the likelihood of achieving a successful outcome from the RCT compared to those experiencing NEF or failure, when other variables such as sex, age, weight, and type of sealer used were adjusted for. Moreover, the 95% credible interval for the number of roots with preexisting PAL ranged from 0.24 to 0.75, denoting statistical significance. Conversely, the 95% credible intervals for all other variables indicated a lack of statistical significance. Consequently, the impact of the different sealers on RCT outcomes was not statistically significant (Table 4).

**Table 2:** Outcomes of RCT in different weight groups.

Group by weight	Number (*N* = 120)	RCT results		
		Success (*n* = 109)	NEF (*n* = 10)	Failure (*n* = 1)
2.5–5 kg	19	18	0	1
5.1–10 kg	57	52	5	0
10.1–15 kg	33	29	4	0
15.1–20 kg	9	8	1	0
20.1–24.5 kg	2	2	0	0

RCT, root canal treatment; NEF, no evidence of failure.

**Table 3:** Comparison of outcome between overall RCT and RCT of the teeth with preexisting PAL.

Sealer (Number of teeth)	Outcome	Number of teeth	Number of teeth with preexisting PAL	Number of teeth	Root number with preexisting PAL		
					One	Two	Three
Silicone-based sealer (67)	Success	62	7	2	2	0	0
	NEF	5		5	5	0	0
	Failure	0		0	0	0	0
Bioceramic sealer (35)	Success	29	17	11	4	3	4
	NEF	5		5	3	1	1
	Failure	1		1	0	1	0
Calcium hydroxide-based sealer (18)	Success	18	0	0	0	0	0
	NEF	0		0	0	0	0
	Failure	0		0	0	0	0
Total (120)	Success: 109 (90.83%) NEF: 10 (8.33%) Failure: 1 (0.83%)		Total (24)	Success: 13 (54.16%) NEF: 10 (33.33%) Failure: 1 (4.16%)			

RCT, root canal treatment; PAL, periapical lesion; NEF, no evidence of failure.

**Table 4:** Posterior distribution of cumulative odds ratio calculated from coefficient estimates of multivariable ordinal regression on outcomes of RCT.

Variable		Cumulative odds ratio		
		Mean	95% credible intervals	
			Lower	Upper
Sex (Ref. = female)		1.70	0.78	3.56
Age		1.03	0.78	1.38
Number of roots having preexisting PAL		0.43	0.24	0.75
Weights	2.5–5 kg	4.59	0.54	45.53
	5.1–10 kg	3.23	0.45	23.57
	10.1–15 kg	2.32	0.39	17.99
	15.1–20 kg	3.04	0.31	37.71
	20.1–24.5 kg	2.66	0.10	89.12
Sealer	Silicone-based sealer	1.40	0.38	5.31
	Bioceramic sealer	1.30	0.33	5.16
	Calcium hydroxide-based sealer	2.34	0.51	12.43

RCT, root canal treatment; Ref., reference; PAL, periapical lesion.

**Figure 3:**
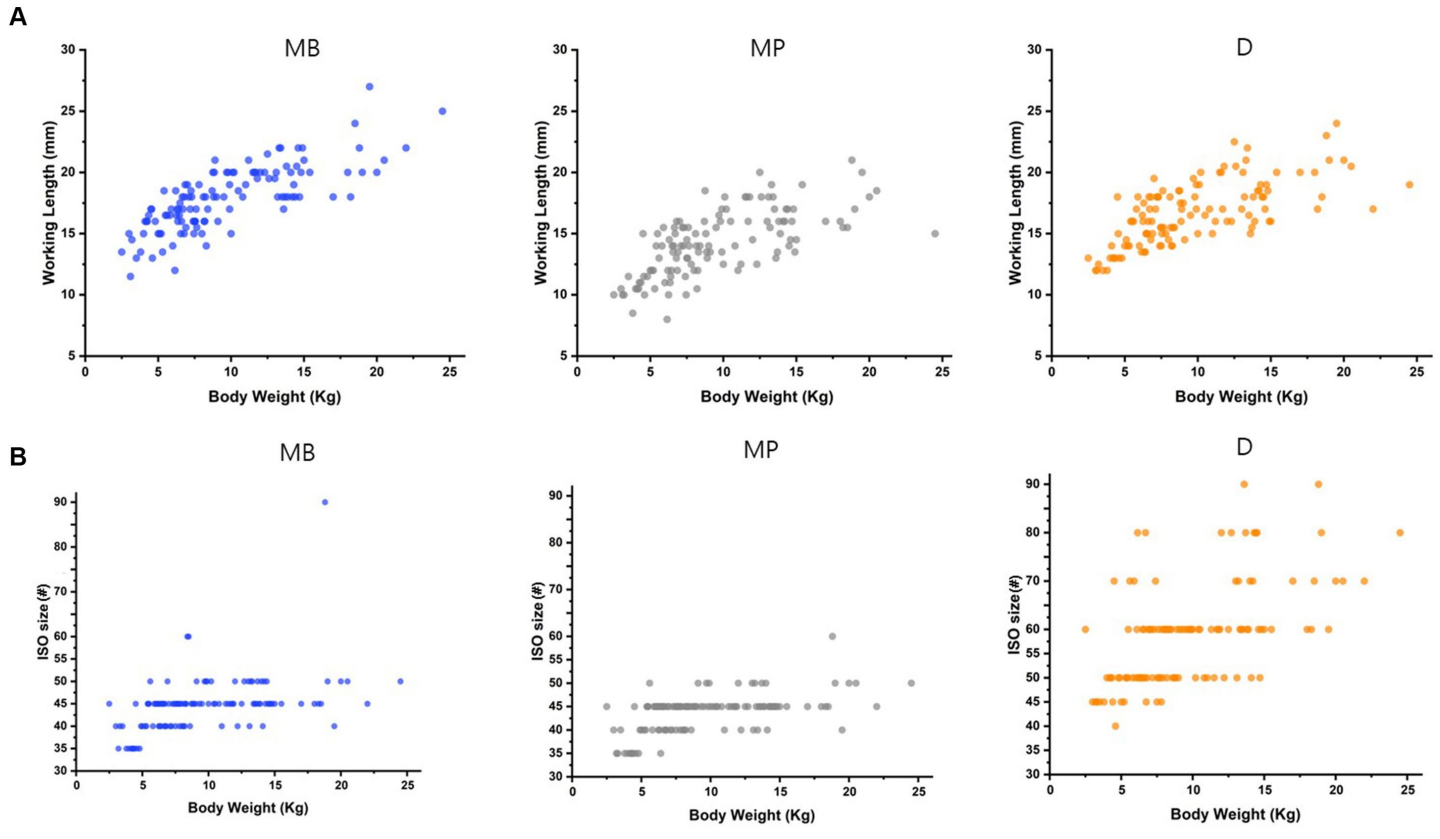
Scatter plots for WL and MAF size of each of the three roots. **(A)** Distribution of working length (WL) per body weight at each root. **(B)** Scatter plots of the master apical file (MAF) size per weight at each root.

The WL and MAF size characteristics of each root are delineated (Table 5; Figure 3). The average WL was 17.89 mm for the mesiobuccal root (11.5–27 mm), 14.15 mm for the mesiopalatal root (8–20 mm), and 16.25 mm for the distal root (12–22.5 mm). The average MAF sizes of the mesiobuccal, mesiopalatal, and distal roots were 45, 45, and 60, respectively. The longest recorded working length among all roots was 27 mm in the mesiobuccal root and the largest MAF size was 90 in the distal root (Figure 3).

**Table 5:** Mean WL and MAF size in each root of maxillary fourth premolars in 120 patients.

Variable (Unit)		Mean ± SD
WL	MB (mm)	17.89 ± 2.62
	MP (mm)	14.15 ± 2.81
	D (mm)	16.25 ± 3.21
MAF size	MB (Iso size)	#45^a^
	MP (Iso size)	#45^b^
	D (Iso size)	#60^c^

a: 0.45 ± 0.06 (mm), b: 0.43 ± 0.05 (mm), c: 0.59 ± 0.11 (mm).

WL, working length; MAF, master apical file; SD, standard deviation; MB, mesiobuccal root; MP, mesiopalatal root; D, distal root.

The WL and MAF size trends for each root were represented as distribution plots in the context of a weight-based group (Figure 3). We observed that the WL of each root tended to increase with increasing body weight; however, a somewhat shorter WL for the mesiopalatal and distal roots was noted in the 20.1–24.5 kg weight group as compared to the 15.1–20 kg group (Figure 3). Furthermore, the MAF size of each root continuously increased as body weight increased. However, notable variations in size were observed among the different weight groups, particularly in the distal root when compared to the other root types (Figure 3).

## 4 Discussion

The purpose of RCT is to remove infected pulp, eliminate bacteria in the pulp cavity, and fill the root canal. The treatment commonly consists of cleaning, shaping, obturation, and sealing of the pulp cavity (10–13, 15, 18–22). In this study, root canals shaped with an engine-driven rotary Ni-Ti file system and treated with a single-cone technique had a 90.83% success rate, and when NEF was included, it was very high at 99.16%. Kuntsi-Vaattovaara et al. reported that the overall success rate of RCT in dogs was 69% (95% including NEF), suggesting that RCT could be a viable option for the salvage of periodontally sound but endodontically diseased teeth in dogs (8). Lee et al. described the overall success rate of RCT in dogs as 71% (96% including NEF), which is similar to previous studies. Moreover, the literature suggests that RCT of complicated fractured canine teeth in dogs should be recommended over extraction because of better long-term outcome (9, 11–13, 23). This finding was reaffirmed in 2022 in a study by Adrian et al., which confirmed the high utility of RCT for fractured canine teeth (success rate 92.73% and NEF 5.45%) (4). However, Kuntsi-Vaattovaara et al. reported that the success rate of RCT for canine teeth was lower than that of maxillary fourth premolars (56% versus 78%, respectively) (8).

In a recent article by Jucan et al., who examined the outcome of an RCT on 45 incisors and reported a high success rate of 93.3% (100% including NEF), the uncomplicated endodontic system of the incisors was suggested as the reason for the high success rate (24). Lee et al. reported a relatively low RCT success rate for maxillary fourth premolars and mandibular first molars, which have relatively complex endodontic systems (9). However, this result was not statistically significant due to the limited number of teeth analyzed. Our study analyzed the RCT outcomes of 120 maxillary fourth premolars and demonstrated a similarly high success rate compared to other studies that reported success rates for canine and incisor teeth in dogs.

The success and prognosis of RCT in dogs and humans are affected by the obturation method used, bacterial penetration in the root canal related to incorrect coronal restoration (25), overfilling of filling materials (26–29), presence of preexisting PAL (30–32), and inadequate obturation of the root canal (31, 33). Preexisting PAL, preoperative pulp necrosis, and EIRR have been shown to decrease success rate (4, 8). In addition, the existence, size, and location of the void and the presence or absence of overfill did not affect the results in previous canine studies (4, 8). Unlike earlier studies in humans and dogs, Lee et al. suggested that preexisting PAL and preoperative EIRR did not significantly influence treatment outcomes in RCT (9). In contrast to the finding of Lee et al., our study confirmed the preexisting PAL was the leading cause of decreased endodontic success. A higher number of preexisting PALs in the maxillary fourth premolars is associated with increased odds of failure rather than success of RCT. In our study, the effect of EIRR was not evaluated because preoperative EIRR cases were not selected as candidates for RCT, particularly when they had preexisting PAL. Cases with preoperative EIRR, a major factor in the failure rate of RCT (4, 8), and CCRF with more than 4 mm pockets, which cannot maintain a sound periodontium, were excluded from RCT in our study. This exclusion may explain the high overall success rate in our study.

In a human clinical study, a thermoplasticized GP system filled more than 95% of the root canal space with GP, and cold lateral compaction sealed more than 80% of the space with GP. In comparison, the single-cone technique was described as filling relatively less space in the canal with GP, and its success rate was 4–5% lower than that of other obturation methods (15). If the GP closest to the size of the shaped root canal is chosen, most of the root canal, especially the apical third, can be filled by a GP with minimal sealer. In the case of canine teeth with relatively long root canals, shaping the canal using zero-tapered files may be advantageous for preserving the peri-cervical dentin. However, in the case of the maxillary fourth premolar teeth in small-to medium-sized dogs with relatively short root canals, applying the single-cone technique using GP that fits the size and shape of the root canal following canal shaping with a tapered file system is beneficial. In our study, the success rate of RCT of fractured maxillary fourth premolars using single-cone techniques was very high (90.83%) compared to the success rate of maxillary fourth premolars (39%) published by Lee et al. (9).

Our results indicated that the type of sealer used in the single-cone technique did not significantly affect the treatment outcomes. The calcium hydroxide-based sealer applied to 18 teeth had a 100% success rate, and the silicone-based sealer applied to 67 teeth had a 92.53% success rate, whereas 29 (82.85%) teeth were evaluated as successful from the 35 teeth in which bioceramic sealer was used; five teeth were assessed as NEF (14.28%) and one root failed (2.85%). At our institution, bioceramic sealers are mainly used when the size of the preexisting PAL is somewhat large or when the condition of the root canal is not considered favorable for RCT success (based on the veterinarian’s experience), such as in older non-vital teeth with weak dentin structure. Bioceramic sealer was applied to 17 teeth with preexisting PAL, and the treatment success rate was 64.70%, whereas the success rate in seven teeth with preexisting PAL treated with silicone-based sealer was 28.57%. As a calcium silicate-based cement, bioceramic sealer has several clinical applications, including pulp capping, pulpotomy, apexogenesis, apexification, perforation repair, and root-end filling, owing to its excellent sealing properties (34), antibacterial effects, biocompatibility (35), and new cement formation (36). Holland et al. determined that the tooth apex was closed with new cement formation after the use of a histologically efficacious bioceramic sealant with a GP cone in canines (37, 38). Bernabe et al. retrofilled pulp teeth in a dog and determined that bioceramic sealer stimulated hard tissue deposition in the open apex compared to other sealants (39). Furthermore, Yildirim and Gencoglu reported positive results in humans with the application of a bioceramic sealer in clinical cases with large PAL (40).

Changes in periapical periodontitis after RCT in dogs have been compared using conventional intraoral radiography and cone beam computed tomography (CBCT) (41, 42). The evaluation of CBCT scans of periapical repair after RCT provided information similar to that obtained from microscopic analysis, whereas radiographic evaluation underestimated the size of the PAL (41). The detection rate of PAL using CBCT (79%) is almost twice as high as that using conventional intraoral radiography (35%) (42). Based on these findings, CBCT may provide more accurate assessments of the success of RCTs. Furthermore, de Paula-Silva et al. established that the mean area of periapical disease on CBCT was more mesiodistally extended than that on conventional radiography at 6 months post-RCT (41). When this is considered, a first follow-up of at least 6 months after the RCT would be appropriate. The mean follow-up duration in the present study was 11.65 months, and the earliest time to follow-up was 3 months.

The MAF sizes and WL of the three roots of 120 teeth in dogs (under 11 years of age and 25 kg body weight) in our study were analyzed. In more than half the cases, the MAF size of the mesiobuccal root of the maxillary fourth premolar was 45 (61 roots). For the mesiopalatal roots, 45 was also the most common MAF size (69 roots), while for the distal roots, 60 was the most frequent MAF size (45 roots). The WL of the three roots did not exceed 31 mm. Although the measured range has been studied to some extent (43), the differences in human tooth size according to race, sex, and body type are insignificant. Accordingly, 21-, 25-, 28-, and 31-mm file systems are the most commonly commercialized systems in human dentistry. Unlike humans, the teeth of dogs vary in size. To the best of our knowledge, no studies have investigated and categorized differences in the size of specific teeth in dogs according to factors such as age, breed, and weight. The present analysis of the MAF size and WL of each of the three roots of the maxillary fourth premolars, which indicated a high success rate in the first RCT of dogs weighing <25 kg, may provide a reference endodontic veterinarians.

The obturation materials used and the quality of obturation do not affect the outcome of canine RCTs (4, 8, 9, 23), and Jucan et al. suspected that other factors might have an impact (24) and we confirmed this suggestion. However, it is possible that the outcomes of the previous studies were achieved because the RCTs were performed by an expert following a precise procedure, with canal scouting to ensure correct WL and adequate shaping and irrigation. If any of these steps is incorrect, the results may not be accurate.

In our study, the root canal was shaped using the engine-driven rotary Ni-Ti file system, and then the canal was obturated with a single GP cone of corresponding size using a minimal sealer with the single-cone technique. This produced a very high treatment success rate. Therefore, we suggest that RCT of the maxillary fourth premolars in small-to medium-sized dogs is an effective option for maintaining tooth function. In addition, there was no significant difference in the success rate depending on the type of sealer.

## 5 Conclusion

In this retrospective study, the results of RCT performed on 120 fractured maxillary fourth premolar teeth in dogs were analyzed. RCT was performed using a silicone-based, bioceramic, or calcium hydroxide-based sealer, with no statistically significant difference in the outcome. The WL and MAF size data for small-to medium-sized dogs collected in this study may be useful for future veterinary use.

## Data availability statement

The raw data supporting the conclusions of this article will be made available by the authors, without undue reservation.

## Ethics statement

Ethical approval was not required for the studies involving animals in accordance with the local legislation and institutional requirements because as this was a retrospective study, approval of IACUC (or similar) was not needed. Written informed consent was obtained from the owners for the participation of their animals in this study.

## Author contributions

DK: Conceptualization, Data curation, Formal analysis, Investigation, Methodology, Visualization, Writing – original draft. DY: Data curation, Formal analysis, Resources, Visualization, Writing – review & editing. SKa: Investigation, Validation, Writing – review & editing. KJ: Conceptualization, Formal analysis, Methodology, Validation, Writing – review & editing. SKi: Conceptualization, Funding acquisition, Methodology, Project administration, Supervision, Validation, Writing – review & editing.
